# Analysis of drug binding pockets and repurposing opportunities for twelve essential enzymes of ESKAPE pathogens

**DOI:** 10.7717/peerj.3765

**Published:** 2017-09-19

**Authors:** Sadia Naz, Tony Ngo, Umar Farooq, Ruben Abagyan

**Affiliations:** 1 Department of Chemistry, COMSATS Intitute of Information Technology, Abbottabad, Pakistan; 2 Skaggs School of Pharmacy & Pharmaceutical Sciences, University of California, San Diego, CA, United States of America; 3 Molecular Cardiology and Biophysics Division, Victor Chang Cardiac Research Institute, Darlinghurst, NSW, Australia

**Keywords:** ESKAPE Pathogens, Sequence alignment, Identity maps

## Abstract

**Background:**

The rapid increase in antibiotic resistance by various bacterial pathogens underlies the significance of developing new therapies and exploring different drug targets. A fraction of bacterial pathogens abbreviated as ESKAPE by the European Center for Disease Prevention and Control have been considered a major threat due to the rise in nosocomial infections. Here, we compared putative drug binding pockets of twelve essential and mostly conserved metabolic enzymes in numerous bacterial pathogens including those of the ESKAPE group and *Mycobacterium tuberculosis*. The comparative analysis will provide guidelines for the likelihood of transferability of the inhibitors from one species to another.

**Methods:**

Nine bacterial species including six ESKAPE pathogens, *Mycobacterium tuberculosis* along with *Mycobacterium smegmatis* and *Eschershia coli*, two non-pathogenic bacteria, have been selected for drug binding pocket analysis of twelve essential enzymes. The amino acid sequences were obtained from Uniprot, aligned using ICM v3.8-4a and matched against the Pocketome encyclopedia. We used known co-crystal structures of selected target enzyme orthologs to evaluate the location of their active sites and binding pockets and to calculate a matrix of pairwise sequence identities across each target enzyme across the different species. This was used to generate sequence maps.

**Results:**

High sequence identity of enzyme binding pockets, derived from experimentally determined co-crystallized structures, was observed among various species. Comparison at both full sequence level and for drug binding pockets of key metabolic enzymes showed that binding pockets are highly conserved (sequence similarity up to 100%) among various ESKAPE pathogens as well as *Mycobacterium tuberculosis*. Enzymes orthologs having conserved binding sites may have potential to interact with inhibitors in similar way and might be helpful for design of similar class of inhibitors for a particular species. The derived pocket alignments and distance-based maps provide guidelines for drug discovery and repurposing. In addition they also provide recommendations for the relevant model bacteria that may be used for initial drug testing.

**Discussion:**

Comparing ligand binding sites through sequence identity calculation could be an effective approach to identify conserved orthologs as drug binding pockets have shown higher level of conservation among various species. By using this approach we could avoid the problems associated with full sequence comparison. We identified essential metabolic enzymes among ESKAPE pathogens that share high sequence identity in their putative drug binding pockets (up to 100%), of which known inhibitors can potentially antagonize these identical pockets in the various species in a similar manner.

## Introduction

The European Centre for Disease Prevention and Control (ECDC) as well as The Infectious Diseases Society of America (IDSA) have recently termed a set of difficult-to-treat bacterial pathogens, *Enterococcus faecium*, *Staphylococcus aureus*, *Klebsiella pneumoniae*, *Acinetobacter baumannii*, *Pseudomonas aeruginosa* and *Enterobacter s* pp, as “ESKAPE” pathogens given their propensity to develop resistance and ‘escape’ from current antibiotics, leading to an increase of nosocomial infections in hospitalized patients (Boucher et al., 2009; Rice, 2008; Spellberg et al., 2008). Infections by methicillin-resistant *S. aureus* (MRSA), multidrug-resistant *P. aeruginosa*, and Vancomycin resistant *E. faecium* as well as various strains of *Mycobacterium tuberculosis* causing multi-drug resistant tuberculosis (MDR-TB) are a global health threat (Klevens et al., 2006; Pomerantz et al., 2001; NNIS, 2004). To address this urgent and unmet medical need, new drugs acting on previously unexplored targets need to be developed.

**Figure 1:**
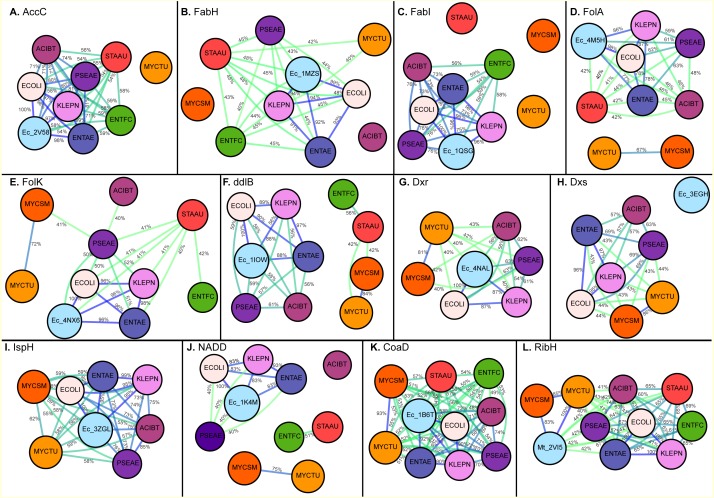
Maps generated for full sequence identity calculation (A) Acetyl-coenzyme A carboxyl transferase beta chain (**AccC**), (B) Beta-ketoacyl-acyl carrier protein synthase III (**FabH**), (C) Enoyl-[acyl-carrier-protein] reductase [NADH] (**FabI**), (D) Dihydrofolate reductase (**FolA**), (E) 2-amino-4-hydroxy-6-hydroxymethyldihydropteridine pyrophosphokinase (**FolK**), (F) D-alanine–D-alanine ligase (**ddlB**), (G) 1-deoxy-D-xylulose 5-phosphate reductoisomerase (**Dxr**), (H) 1-deoxy-D-xylulose 5-phosphate synthase (**Dxs)**, (I) 4-hydroxy-3-methylbut-2-enyl diphosphate reductase (**IspH**), (J) Nicotinate-nucleotide adenylyltransferase (**NADD**), (K) Phosphopantetheine adenylyltransferase (**CoaD**), (L) 6,7-dimethyl-8-ribityllumazine synthase (**RibH**). The edges are colored with a green (low sequence) to blue (high sequence identity) gradient, where sequence identities between two nodes below 40% are not shown.

Although various enzymatic targets involved in bacterial metabolic pathways have been investigated for antibiotic discovery, a large proportion of them remain unexplored: a genomics-based selection, prioritization and a list of approximately one hundred essential metabolic enzymes conserved across numerous diverse bacterial pathogens have been revealed as potential new targets (Osterman & Begley, 2007; Payne et al., 2007). The degree of conservation among orthologous sequences vary throughout the sequence and usually have low sequence similarity on a full sequence alignment level as depicted in Fig. 1. However, emphasis should be put on the residues involved in chemical interactions, that is, residues forming the drug binding pocket given that slight variation in amino acids of drug binding pockets responsible for enzymatic activity can cause considerable changes in substrate and/or drug binding (Tytgat et al., 2009). We have previously compared drug-binding pockets of various enzymes orthologs through sequence alignment and have been successful in identifying new off-targets for receptors (McRobb et al., 2014; Ngo et al., 2017). Species having conserved active sites might interact with inhibitors in similar way that can be helpful for identification of inhibitors for diseases caused by the various species. For illustration, the effect of active inhibitors on DapL orthologs like *Arabidopsis thaliana* (AtDapL), a flowering plant, *Leptospira interrogans* (LiDapL), the soil/water bacterium *Verrucomicrobium spinosum* (VsDapL) and the alga *Chlamydomonas reinhardtii* (CrDapL) have been previously reported and it was found that AtDapL and LiDapL showed the same trend of inhibition for different inhibitors in study. It clearly suggests that inhibitors effective against one species may have potential of being active for other species as well (McKinnie et al., 2014).

**Table 1:** Enzymes, their abbreviations, their human counter targets, reference PDB used for analysis and inhibitors for twelve enzyme targets.

S.No	Enzyme	Abb	Human counter targets		Ref PDB	Substrates or compounds/ Species^a^
			Presence	PDB		
1	Acetyl-coenzyme A carboxyl transferase beta chain	AccC	Distant homolog: domain of FasN	1XKT (FASN), 5I87 (Acc2)	2V58 *E. coli*	Biotin, Adenine
2	Beta-ketoacyl-acyl carrier protein synthase III	FabH	Distant homolog: domain of FasN	NA	1MZS *E. coli*	Ceruleninpar (*Saccharomyces cerevisiae*)
3	Enoyl-[acyl-carrier-protein] reductase [NADH]	FabI	None	NA	1QSG *E. coli*	Triclosan (*Staphlococcus aureus*)
4	Dihydrofolate reductase	Fol A	Distant homolog of DHFR	4QHV	4NX6 *E. coli*	Isoniazid (*M. tuberculosis)*, Proguanil (*Plasmodium falciparum*) Methotrexate
5	2-amino-4-hydroxy-6-hydroxymethyldihydropteridine pyrophosphokinase	Fol K	None	NA	4M5H *E. coli*	Aminosalicylic acid (*M. tuberculosis*)
6	1-deoxy-D-xylulose 5-phosphate synthase	Dxs	None	NA	2EGH *E. coli*	–
7	4-hydroxy-3-methylbut-2-enyl diphosphate reductase	IspH	None	NA	3ZGL *E. coli*	–
8	1-deoxy-D-xylulose 5-phosphate reductoisomerase	Dxr	None	NA	4NAL *E. coli*	–
9	D-alanine–D-alanine ligase	ddlB	None	NA	1IOW *E. coli*	Cycloserine (*M. tuberculosis*)
10	6,7-dimethyl-8-ribityllumazine synthase	ribH	None	NA	2VI5 *M.tuberculosis*	–
11	Nicotinate-nucleotide adenylyltransferase	NADD	Distant homolog, NMNAT	1KR2	1K4M *E. coli*	Niacin
12	Phosphopantetheine adenylyltransferase	CoaD	Distant homolog domain of PPAT/DPCK bifunctional enzyme	3DO8 (Closer homolog of Human)	1B6T *E. coli*	–

**Notes.**

Abb, abbreviation.

a As found in Drugbank.

Since binding sites of enzymes from different species are generally more conserved than their overall structures (Bartlett et al., 2002; Caffrey et al., 2004), we compared the drug binding pockets of the key enzymes involved in selected essential metabolic pathways conserved across the ESKAPE pathogens, *M. tuberculosis*, and other pathogenic and model bacteria using experimentally determined crystal structures of representative orthologs and amino acid sequence alignments. The majority of these selected enzymes lack human orthologs (or close homologs), which is important for antibiotic discovery to minimize side-effects via inhibition of respective host enzymes (Table 1). However this criteria is not always absolute, as some antibiotics target specifically bacterial enzymes with sufficient selectivity and have nearly no inhibitory effects on respective human ortholog. For example, Trimethoprim is more potent inhibitor of bacterial dihydrofolate reductase and have very weak inhibitory activity for human DHFR although its human ortholog share 28% sequence identity (Sarker et al., 2012; Schweitzer, Dicker & Bertino, 1990). The binding pocket residues of the relevant enzymes, defined by experimental crystal structures captured in the Pocketome encyclopedia (Kufareva, Ilatovskiy & Abagyan, 2011), were propagated onto a master alignment of the enzyme across different bacterial species.

The results of this comparative analysis showed that specific enzymatic binding pockets are highly identical (with sequence identity in the range of 70–100%) across some of the ESKAPE pathogens suggesting that inhibitors designed to target one enzyme from a particular bacterial species may have potential activity targeting the same enzyme in a different species. In addition, this approach also enables selection of the most appropriate model bacterial pathogens as a proxy for early stages of drug development and assists in drug repurposing.

## Material and Methods

### Selection of pathogens

Nine different bacterial pathogens were selected including six ESKAPE pathogens, namely *Enterococcus faecium*, *Staphylococcus aureus*, *Klebsiella pneumoniae*, *Acinetobacter baumannii*, *Pseudomonas aeruginosa* and *Enterobacter aurogenes*. *Mycobacterium tuberculosis* and two model non-pathogenic bacterial species *Mycobacterium smegmatis* and *Escherichia coli* were also included in our binding pocket comparative analysis.

### Selection of essential enzyme targets

Twelve essential and mostly conserved metabolic enzymes were selected as priority targets for our study from a larger set of high-ranked metabolic drug targets based on previous comparative and functional genomics analyses (Osterman & Begley, 2007) (Table 1). These critical enzymes catalyze key steps in various metabolic pathways including folate biosynthesis, fatty acid biosynthesis, isoprenoid biosynthesis, peptidoglycan biosynthesis, nicotinamide adenine dinucleotide (NAD) and NADH biosynthesis, and riboflavin metabolism. More importantly, our target selection was driven by the availability of experimentally determined 3D structures and complexes of the respective protein target, independent of the species. In addition to other criteria, the majority of these selected enzymes lack human orthologs (or close homologs), which is important for antibiotic discovery to minimize side effects via inhibition of respective host enzymes (Table 1).

### Uniprot sequences and their alignment

The amino acid sequences of the selected enzymes for each of the respective bacterial pathogens were obtained from the Uniprot database (The UniProt Consortium, 2017). The sequences for each enzyme were then aligned using ICM v3.8-4a or above (Molsoft L.L.C., La Jolla, CA) (Abagyan & Totrov, 1994). The selected enzyme targets were matched against the Pocketome encyclopedia (Kufareva, Ilatovskiy & Abagyan, 2011), which contains a set of collected and annotated binding pocket structures derived from the Protein Data Bank (PDB), allowing accurate, experimentally-derived definitions of their respective binding pockets used for this analysis (Table 1).

### Generation of distance-based maps for binding pocket sequence identity comparison

Based on the full protein sequence, the sequence identity between each of the target enzymes across each species was calculated; sequence identity was measured as identical residue/total number of aligned residues, transformed by the Dayhoff correction and distance-based maps were generated in ICM. The binding pocket of each enzyme was determined by the residue contacts made by small molecule inhibitors co-crystallized with an enzyme from one of the selected pathogens. A subselection of the full sequence alignment was extracted by propagating the determined binding pocket residues over orthologous protein sequences of the selected pathogens. This followed binding pocket identity calculations and regeneration of sequence maps based on the binding site as described above.

The labels used for various microorganisms were as followed: ENTFC for *Enterococcus faecium*, STAAU for *Staphylococcus aureus*, KLEPN for *Klebsiella pneumoniae*, ACIBT for *Acinetobacter baumannii*, PSEAE for *Pseudomonas aeruginosa*, ENTAE for *Enterobacter aurogenes*, MYCTU for *Mycobacterium tuberculosis*, MYCSM for *Mycobacterium smegmatis* and ECOLI for *Escherichia coli*.

### Generation of homology model

A homology model was generated for the enzyme 1-deoxy-D-xylulose 5-phosphate reductoisomerase (Dxr) from *P. aeruginosa* for predictive binding of known inhibitor fosmidomycin into the binding pocket. The crystal structure of Dxr from *Mycobacterium tuberculosis* (PDB ID: 4AIC) was used as the template (45% sequence identity). This model was built by using the ICM homology modeling module.

## Results

### Drug binding pocket conservation of enzymes involved in fatty acid biosynthesis

**Figure 2:**
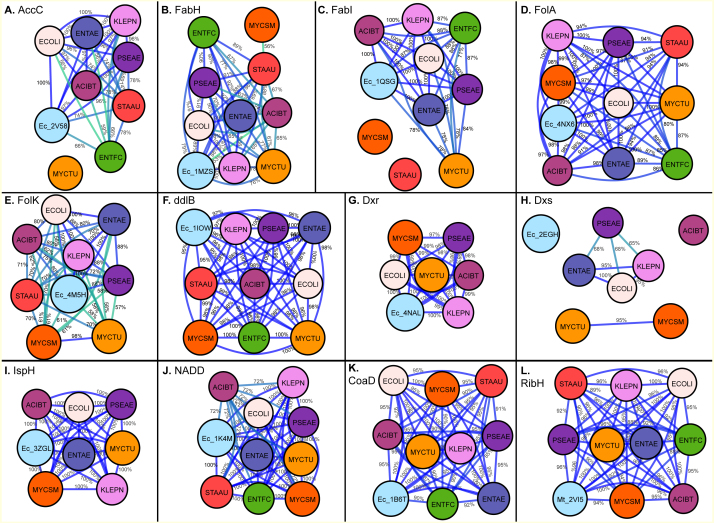
Maps generated for Drug Binding Pocket sequence identity calculation for (A) Acetyl-coenzyme A carboxyl transferase beta chain (**AccC**), (B) Beta-ketoacyl-acyl carrier protein synthase III (**FabH**), (C) Enoyl-[acyl-carrier-protein] reductase [NADH] (**FabI**), (D) Dihydrofolate reductase (**FolA**), (E) 2-amino-4-hydroxy-6-hydroxymethyldihydropteridine pyrophosphokinase (**FolK**), (F) D-alanine–D-alanine ligase (**ddlB**), (G) 1-deoxy-D-xylulose 5-phosphate reductoisomerase (**Dxr**), (H) 1-deoxy-D-xylulose 5-phosphate synthase (**Dxs)**, (I) 4-hydroxy-3-methylbut-2-enyl diphosphate reductase (**IspH**), (J) Nicotinate-nucleotide adenylyltransferase (**NADD**), (K) Phosphopantetheine adenylyltransferase (**CoaD**), (L) 6,7-dimethyl-8-ribityllumazine synthase (**RibH**). The edges are colored with a green (low sequence) to blue (high sequence identity) gradient, where sequence identities between two nodes below 50% are not shown.

**Figure 3:**
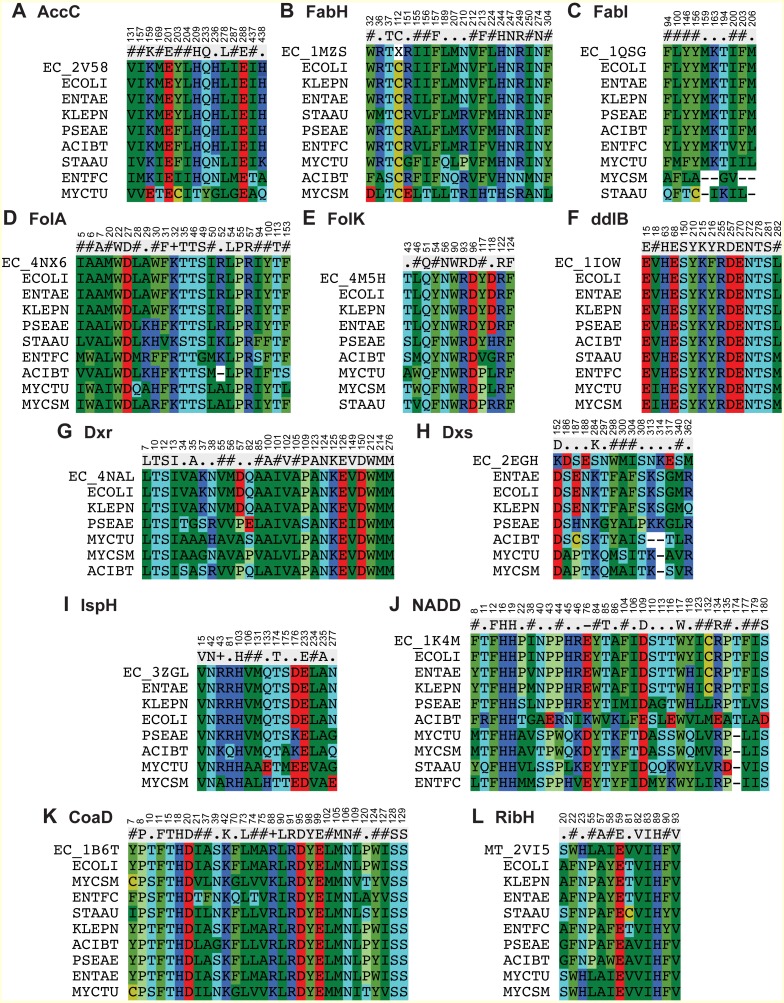
Multiple sequence alignment for drug binding pockets of Enzymes (A) Acetyl-coenzyme A carboxyl transferase beta chain (**AccC**), (B) Beta-ketoacyl-acyl carrier protein synthase III (**FabH**), (C) Enoyl-[acyl-carrier-protein] reductase [NADH] (**FabI**), (D) Dihydrofolate reductase (**FolA**), (E) 2-amino-4-hydroxy-6-hydroxymethyldihydropteridine pyrophosphokinase (**FolK**), (F) D-alanine–D-alanine ligase (**ddlB**), (G) 1-deoxy-D-xylulose 5-phosphate reductoisomerase (**Dxr**), (H) 1-deoxy-D-xylulose 5-phosphate synthase (**Dxs)**, (I) 4-hydroxy-3-methylbut-2-enyl diphosphate reductase (**IspH**), (J) Nicotinate-nucleotide adenylyltransferase (**NADD**), (K) Phosphopantetheine adenylyltransferase (**CoaD**), (L) 6,7-dimethyl-8-ribityllumazine synthase (**RibH**). The residue numbers in the alignments are in reference to the *E. coli* protein.

Acetyl-Coenzyme A carboxylase (AccC) plays a key role in synthesis and degradation of fatty acids. Targeting bacterial acetyl-CoA carboxylase can be an effective approach for discovery of novel antibiotics with low side effects. Of the selected pathogens, acetyl-CoA carboxylase (AccC) from *E. coli* has been crystallized (PDB: 2V58). The comparison of the putative binding pockets based on the AccC *E. coli* crystal structure revealed that binding pocket of AccC from *E. coli* (PDB: 2v58) (Miller et al., 2009) had 100% identity with its ortholog in *E. aurogenes* and *K. pneumoniae* and up to 97% identity in *P. aeruginosa* and *A. baumannii*. In comparison with the full-length sequence, the binding pocket of this enzyme is highly conserved and interestingly, the level of conservation of drug binding pocket is high among Gram-negative species (97–100%) as compared to Gram-positive bacterial species like *S. aureus* and *E. faecium* (up to 74%) as depicted in Figs. 2A and 3A.

Beta-ketoacyl-acyl carrier protein synthase III (FabH) is another key enzyme involve in fatty acid biosynthetic pathway. Using the FabH crystal structure from *E. coli* (PDB: 1MSZ) (Daines et al., 2003), the putative drug binding pocket of *K. pneumoniae* shared 89% identity with the co-crystallized structure whilst the drug binding pockets of FabH among all species in the study have high identity (77–100%) as suggested by the maps (Fig. 2B). Previously, 4,5-dichloro-1,2-dithiole-3-one was reported as potent inhibitor of FabH from *S. aureus* but also in *E. coli*, further supporting our hypothesis that inhibitors effective in one species have potential off target activity against other species (He et al., 2004).

The binding pocket of enoyl-acyl carrier protein reductase (FabI) is highly conserved among various species as compared to the overall sequence. By using the *E. coli* FabI crystal structure (PDB: 1QSG) (Stewart et al., 1999), we found that the respective FabI ortholog putative binding pockets in *E. aurogenes*, *K. pneumoniae*, *P. aeruginosa*, *A. baumannii* were 100% identitical to one another, while sharing greater than 80% sequence identity compared to the putative FabI pocket *E. faecium* and *M. tuberculosis* (Fig. 2C).

### Drug binding pocket conservation of enzymes involved in folate synthesis

The sequence of drug binding pockets of key enzyme dihydrofolate reductase (folA) of folate biosynthesis were found to be highly conserved (81–100%) among various ESKAPE pathogens by using PDB: 4NX6 from *E.coli* for comparison and the same trend was observed in case of 6-Hydroxymethyl-7,8-dihydropterin pyrophosphokinase (folK) except *S. aureus* with reference PDB: 4M5H (Figs. 2D and 2E).

### Drug binding pocket conservation of enzymes involved in isoprenoid biosynthesis

The species *S. aureus* and *E. faecium* involve the mevalonate pathway for isoprenoid biosynthesis so they lack the Dxs, Dxr and IspH enzymes. The amino acid residues involved in ligand interactions of 1-deoxy-D-xylulose 5-phosphate synthase (Dxs) from *E.coli* (PDB: 2EGH) (Yajima et al., 2007) showed sequence identity values below 70% among various bacterial species in current comparison. The other two enzymes of non-mevalonate pathway like IspH and Dxr showed 98–100% conservation of amino acid sequence among all species in comparison based on the crystal structures PDB: 3ZGL and PDB: 3ANL (Deng et al., 2010) from *E.coli* respectively (Figs. 2G–2I).

### Drug binding pocket conservation of enzymes involved in peptidoglycan and CoA biosynthesis

D-alanyl-D-alanine ligase (ddlB) is a significant target for antibiotic discovery as it is essential for bacterial growth. Propagating the drug binding pocket of ddlB from *E.coli* (PDB: 1IOW) (Fan et al., 1997) onto all the other species, it revealed that they all shared 96–100% sequence identity amongst one another as shown in Fig. 2F.

Phosphopantetheine adenylyltransferase (CoaD) enzyme involved in coenzyme A biosynthesis is another attractive target for antibiotic discovery. Similarly, the drug binding pocket of Phosphopantetheine adenylyltransferase based on PDB: 1B6T (Izard & Geerlof, 1999) was found to be highly conserved amongst the ESKAPE, *Mycobacterium tuberculosis* as well as other bacterial species in the range of 85–100% as compared to full sequence (Figs. 1 and 2K).

### Drug binding pocket conservation of NADD and 6,7-dimethyl-8 -ribityllumazine synthase enzyme

The drug binding pocket of co-crystal structure of NADD from *E. coli* (PDB: 1K4M) (Zhang et al., 2002) showed 100% identity with all the species in this comparison, except *A. baumannii*, while the inhibitor binding pocket of the enzyme ribH, which catalyzes a key step in metabolism of Riboflavin resulting in synthesis of flavin mononucleotide (FMN) and flavin adenine dinucleotide (FAD) cofactors, was conserved (90–100%) across the various species as compared to reference binding pocket (PDB: 2VI5) (Zhang et al., 2008) (Figs. 2J and 2L).

**Figure 4:**
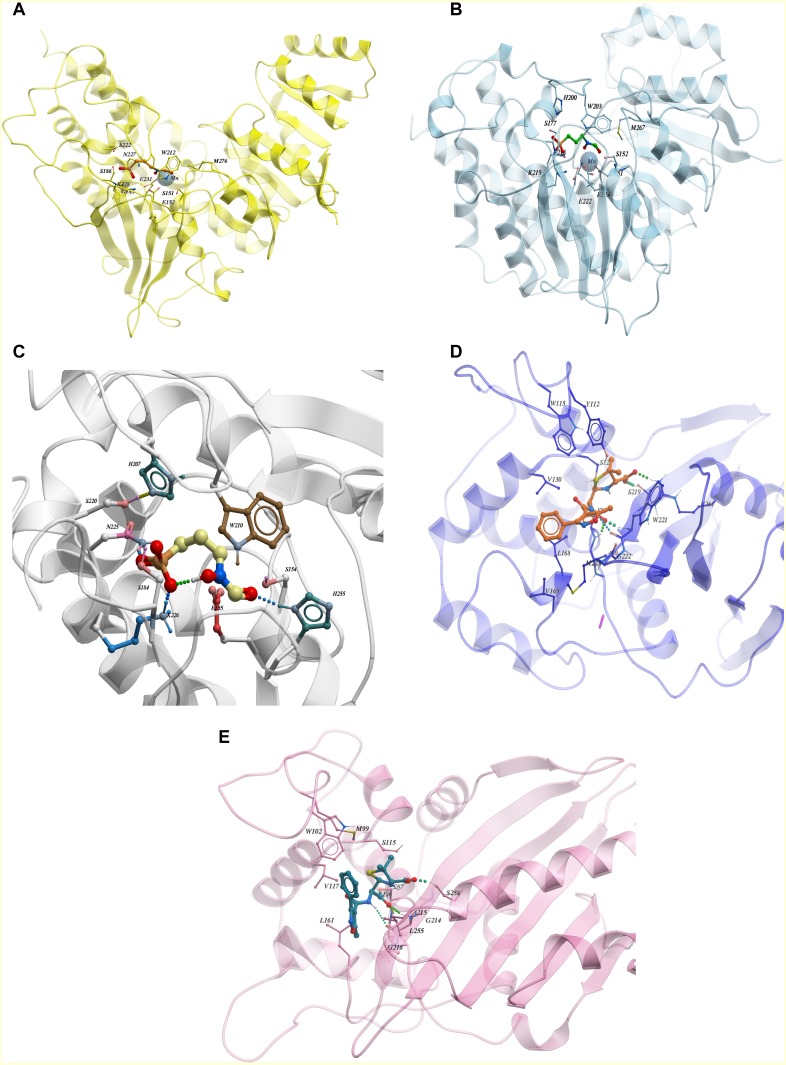
(A) Binding interaction of drug binding pocket of Dxr from *E.coli* (PDB: 1ONP), (B) Binding interaction of drug binding pocket of Dxr from *M. tuberculosis* (PDB: 4AIC), (C) Binding of Fosmidomycin in pocket of model generated for Uniprot sequence of Dxr from *P. aeruginosa*, (D) Binding pocket interaction of oxacillin co-crystallized with *β*-lactamase from *A. baumannii*, (E) Binding pocket interaction of oxacillin co-crystallized with *β*-lactamase from *E. coli*.

### Comparison of fosmidomycin interaction with Dxr binding pockets across various species

From the ChEMBL dataset of active compounds for the enzyme 1-deoxy-D-xylulose 5-phosphate reductoisomerase (Dxr), we found that Fosmidomycin, a natural antibiotic, is an inhibitor for Dxr in both *E.coli* and *M. tuberculosis*. Crystal structures of *E.coli* and *M. tuberculosis* Dxr have been solved with Fosmidomycin (PDB ID: 1ONP and 4AIC respectively) and they display a similar shape and orientation in three-dimensional space (Henriksson et al., 2007; Steinbacher et al., 2003) (Figs. 4A and 4B). In addition, the inhibitor showed similar, if not identical, interactions with binding pocket residues (Fig. 4C); they display 41% sequence identity across the full length sequence, which further strengthens our hypothesis that inhibitors active in one species may have activity for other enzyme orthologs. As Fosmidomycin has shown activity against Dxr of *M. tuberculosis* and *E. coli*, this will help the design of new drugs for tuberculosis by taking advantage of the fosmidomycin chemical scaffold. Fosmidomycin and its derivatives has also been reported as potent inhibitors of Dxr of *P. falciparum*, the causative agent of malaria. To predict its interaction-binding mode, we generated homology model of Dxr from *P. aeruginosa* using PDB: 4AIC as a template and docked Fosmidomycin into the putative binding pocket. The highest ranked docking pose predicted a similar interaction pattern compared to the crystal structures (Fig. 4C). In another example, oxacillin, a β-lactam antibiotic of the penicillin group has been co-crystallized with the Class D β-lactamases oxacillinase OXA-1 from *E. coli* and OXA-24/40 from *A. baumannii* (which shares 99% full length sequence identity with OXA-1) (PDB ID: 4MLL and 4F94, respectively) (June et al., 2014). The comparison of the ligand binding pockets display similar orientation (with slight rotation of an R side chain group between the structures), binding interactions, as well as shape (June et al., 2014) as depicted in Figs. 4D and 4E. The examples of fosmidomycin and oxacillin further demonstrates the idea of conserved binding pockets and cross-species activity.

## Discussion

The current study compared the putative binding pockets of a wide range of broad-spectrum antibacterial targets selected from the set of essential metabolic enzymes, conserved across the group of ESKAPE pathogens. On the assumption that enzymes share a common binding pocket spatially, we propagated experimentally determined drug binding pockets of enzymes of one pathogen onto all other important pathogens by alignment. We performed comparison at the level of full sequence and for drug binding pockets and found that the binding pocket sequences were highly conserved across the ESKAPE pathogens (Fig. 2 and 3). Although the percent sequence identity calculated for full sequence alignment for all these enzymes was still reasonable (Fig. 1), the binding pocket comparison revealed a high degree, if not, full conservation, which suggests that there is potential for off-target activity by these inhibitors and allow the ease of drug repurposing and expansion of disease indications.

Generally, key metabolic enzymes from the ESKAPE pathogens displayed conserved binding pockets (extrapolated from experimentally-determined structures) across various bacterial species. This suggests that inhibitors, which are effective for one species has the potential to be active for other species, providing ligand design cues for broad-range inhibitors. In addition, binding pockets of enzymes including ddlB, CoaD, FabH, FabI, FolA and IspH showed high sequence identity, not only across the ESKAPE pathogens, but also with *M. tuberculosis*, which will ultimately guide the identification of new inhibitors against tuberculosis (Fig. 2). In particular, the binding pocket of IspH showed 100% sequence identity among all species in this comparison (Fig. 2) and, thus, known inhibitors of IspH enzymes is a suitable initial ligand screening set for all bacterial species. There is a precedent, where N-[2,4-Dioxo-6-D-ribitylamino-1,2,3,4-tetrahydropyrimidin-5-yl] oxalamic acid derivatives were screened and inhibited orthologs of enzyme lumazine synthase from *M. tuberculosis, B. subtilis* and *E.coli* (Zhang et al., 2008). Therefore, the concept of taking the same inhibitor for different bacterial species and different diseases is useful for the identification of new antibiotics with off target activity.

There are several limitations of this study that need to be considered. Our method is heavily reliant on the availability of at least one crystal structure of the enzyme of interest for accurate interspecies binding pocket comparison, which excludes many potential drug targets. Furthermore, the method is reliant on good sequence alignment of the various enzyme orthologs and does not consider whether these enzymes have similar functions in their respective bacterial species. These limitations will be addressed in the future with the increasing availability of structural data. The incorporation of spatial information and protein folding in the form of homology models, as we have demonstrated for fosmidomycin interactions with Dxr of *P. aeruginosa*, can be used to extend our analysis in the future. In addition, the sequence maps also showed that the binding site of ESKAPE pathogens are closely related to each other; these are all *in silico* predictions and will require experimental validation, which is beyond the scope of this study. However, we believe this could be a new direction for discovering or repurposing new inhibitors for enzyme targets of different orthologs based on inferred binding pocket identity or similarity. Furthermore, we have also focused on essential enzymes here, whereas the bigger challenge is identifying targets, which are less prone to resistance, which will be investigated in future studies.

## Conclusions

The present investigation demonstrated that putative drug binding pockets of key enzymes of ESKAPE pathogens have a higher degree of conservation than full length sequences, based on the extrapolation of experimentally-determined binding pockets of orthologs. We propose that conserved pocket sequence identity will mean that drugs or ligands interacting at one pocket will display the same inhibition at another and that the same chemical scaffold can be used as the basis of drug design for other enzyme orthologs. This potentially could be an effective approach for design of new antibiotics for ESKAPE pathogens.

## Supplemental Information

10.7717/peerj.3765/supp-1 Supplemental Information 1 Alignment of various enzyme FolA orthologs

10.7717/peerj.3765/supp-2 Supplemental Information 2 Alignment of various IspH enzyme orthologs

10.7717/peerj.3765/supp-3 Supplemental Information 3 Alignment of various CoaD enzyme orthologs

10.7717/peerj.3765/supp-4 Supplemental Information 4 Alignment of various AccC enzyme orthologs

10.7717/peerj.3765/supp-5 Supplemental Information 5 Alignment of various ribH enzyme orthologs

10.7717/peerj.3765/supp-6 Supplemental Information 6 Alignment of various FolK enzyme orthologs

10.7717/peerj.3765/supp-7 Supplemental Information 7 Alignment of various Dxr enzyme orthologs

10.7717/peerj.3765/supp-8 Supplemental Information 8 Alignment of various Dxs enzyme orthologs

10.7717/peerj.3765/supp-9 Supplemental Information 9 Alignment of various ddlB enzyme orthologs

10.7717/peerj.3765/supp-10 Supplemental Information 10 Alignment of various fabH enzyme orthologs

10.7717/peerj.3765/supp-11 Supplemental Information 11 Alignment of various fabI enzyme orthologs

10.7717/peerj.3765/supp-12 Supplemental Information 12 Alignment of various NADD enzyme orthologs

## Additional Information and Declarations

### Data Availability

The following information was supplied regarding data availability: All alignment fasta files used to calculate sequence identities have been included as Supplementary Files.
